# Combination treatment for superior mesenteric artery dissection: therapeutic challenge

**DOI:** 10.1590/1677-5449.210157

**Published:** 2022-03-07

**Authors:** Guilherme Borgo Ficagna, Cristiano do Carmo Galindo, Jean Paulo Niero Mazon, Gustavo Galvan Debiasi, Amanda Bogo Vargas, Laura Sahd Bernz

**Affiliations:** 1 Hospital Maternidade Marieta Konder Bornhausen – HMKB, Itajaí, SC, Brasil.; 2 Universidade do Vale do Itajaí – UNIVALI, Itajaí, SC, Brasil.; 3 Hospital de Clínicas de Porto Alegre – HCPA, Porto Alegre, RS, Brasil.

**Keywords:** superior mesenteric artery, dissection, endarterectomy, angioplasty

## Abstract

Dissection of the superior mesenteric artery is a rare cause of abdominal pain, with a variable clinical picture. It is difficult to diagnose and there is no consensus on treatment options, which range from conservative treatment to open, endovascular, or combination repair. We describe the case of a 45-year-old man with isolated dissection of the superior mesenteric artery and persistent abdominal pain after conservative treatment had been attempted. He underwent open surgical revascularization due to the location and complexity of the dissection. Treatment consisting of endarterectomy, arterioplasty with bovine pericardium patch, and retrograde access to open the mesenteric artery with a stent was successful. Abdominal angina was completely resolved after the condition had stabilized. A combination of open and endovascular approaches should be considered as treatment for cases of isolated complex dissection of the superior mesenteric artery.

## INTRODUCTION

Superior mesenteric artery (SMA) dissection is a rare cause of abdominal pains that is difficult to diagnose.^1^^,^ 2 Computed tomography is the most recognized method to detect the condition, since it will show the lesion in the majority of cases and can also be used for treatment planning.^3^^,^ 4 There are various treatment options for SMA dissection, ranging from conservative treatment to open surgical repair,^5^ endovascular repair, or combination treatments,^6^^,^ 7 depending in the severity of the case. The project was approved by a Research Ethics Committee (CAAE 47854621.0.0000.0120, consolidated opinion number 4.875.653).

## PART I: CLINICAL CASE

**Figure 1:**
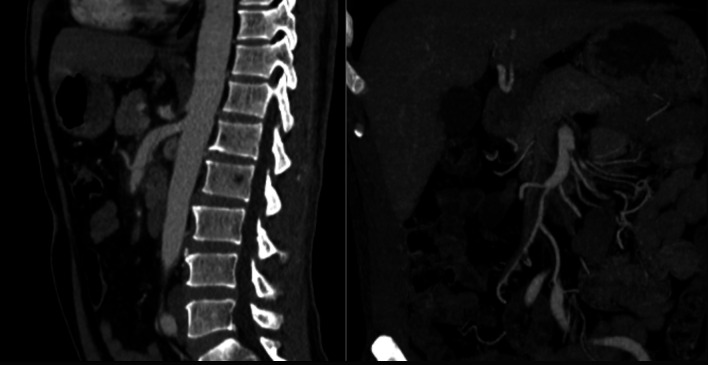
Isolated superior mesenteric artery dissection, with perfusion maintained to all branches.

**Figure 2:**
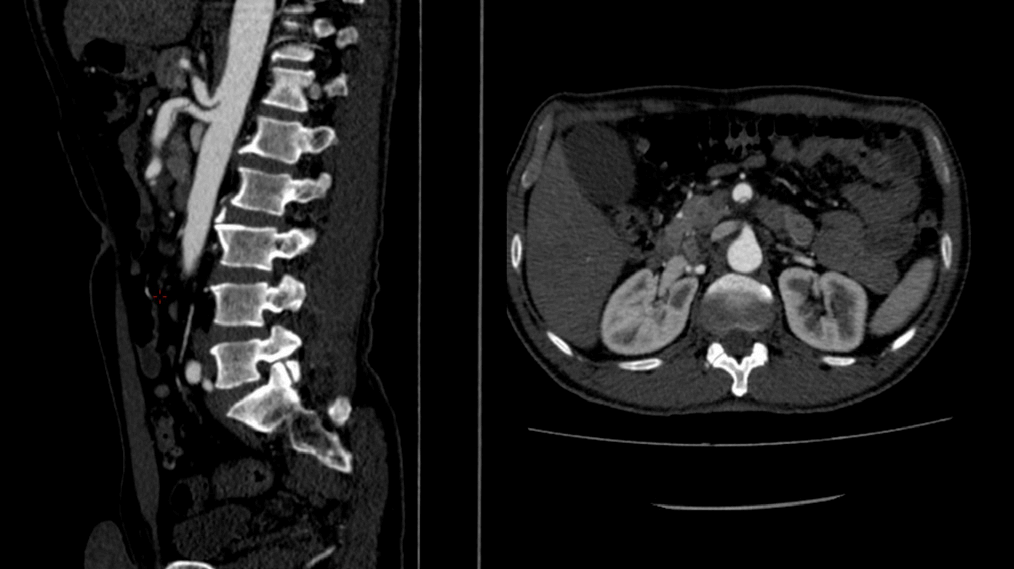
Presence of an intimal flap in the superior mesenteric artery, suggestive of dissection, and absence of vascular flow into the distal branches, suggestive of thromboembolism.

The patient was a 45-year-old, male, hypertensive ex-smoker who presented with diffuse, recurrent abdominal pains with onset 30 days previously, worsening the day before presentation, but with no other complaints. His vital signs were stable and on physical examination of the abdomen he reported mild pain in response to palpation, but no signs of peritoneal irritation. He underwent tomography with contrast, which showed a single dissection of the SMA (Figure 1) with perfusion maintained to all of its branches. At this first contact, the decision was taken to admit him to hospital for symptomatic treatment, with pain control and antiplatelet therapy. Since symptoms improved with analgesia and laboratory test results remained normal throughout his hospital stay, the patient was discharged after 5 days to maintain treatment and attend outpatients follow-up. He returned to the hospital the day after hospital discharge, reporting that his pain had worsened. He underwent tomography with contrast once more, which did not show any changes from the previous examination. He was admitted again because of the pain and discharged 7 days later, clinically asymptomatic and still on antiplatelet therapy. The following day he returned to the hospital because of exacerbation of pain, and control imaging showed absence of vascular flow into the distal branches of the SMA (Figure 2), suggesting thromboembolism. At this point, the next step was to choose a treatment course from among the available options, which were open repair, endovascular repair, or a combination approach.

## PART II: TREATMENT

**Figure 3:**
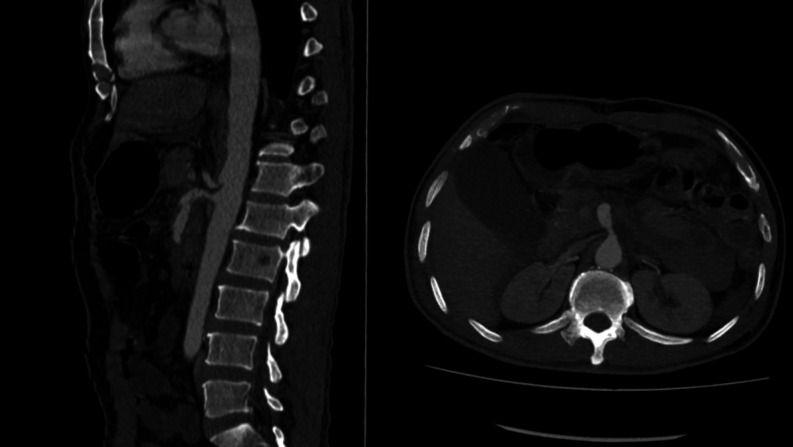
Superior mesenteric artery, patent proximally with abrupt failure to fill the distal portion, suggestive of graft thrombosis.

**Figure 4:**
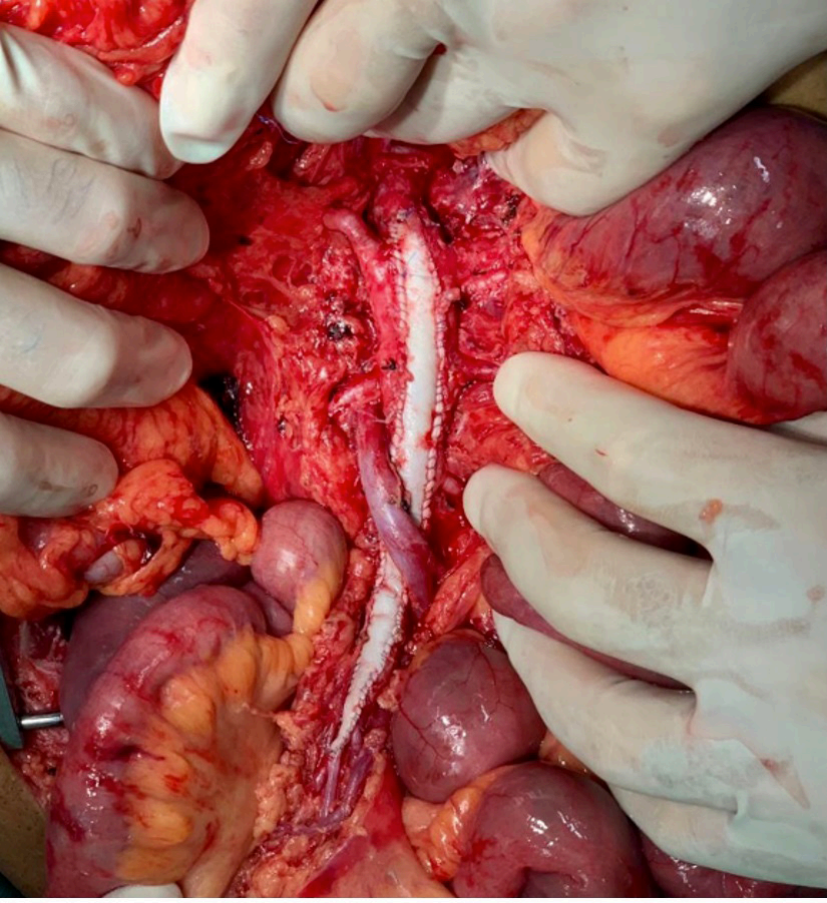
Patched superior mesenteric artery.

**Figure 5:**
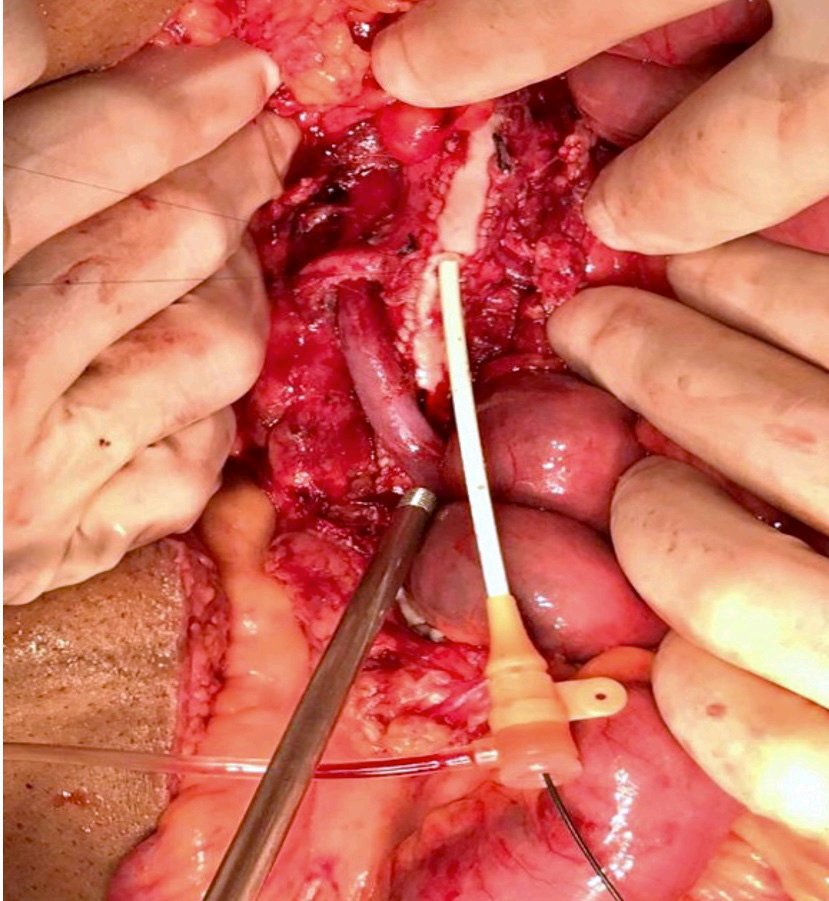
Retrograde puncture via the patch into the superior mesenteric artery.

**Figure 6:**
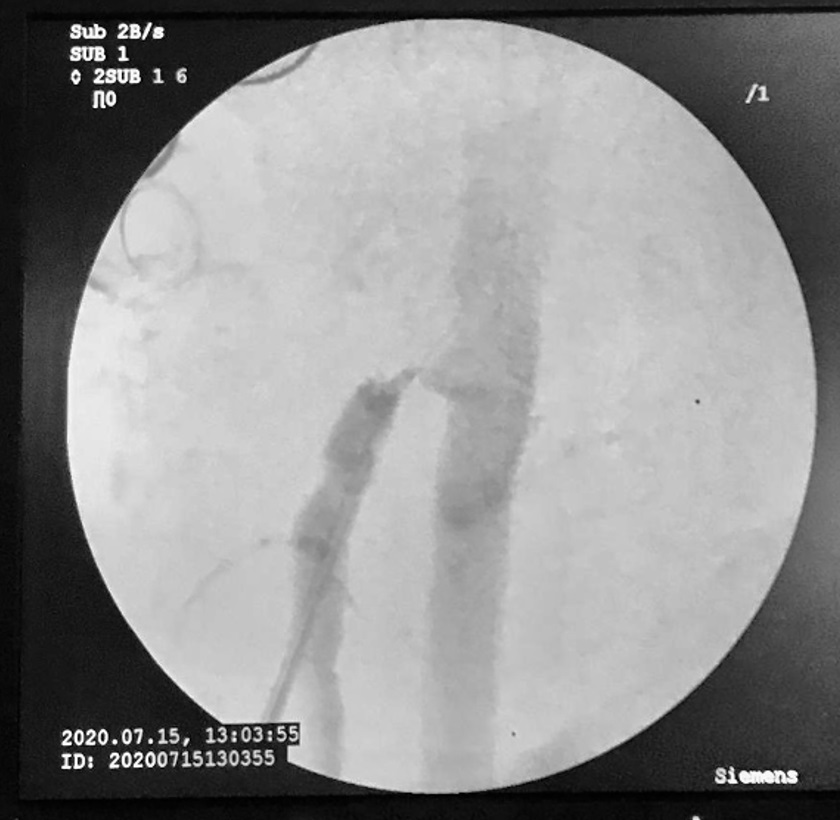
Arteriography of the superior mesenteric artery.

**Figure 7:**
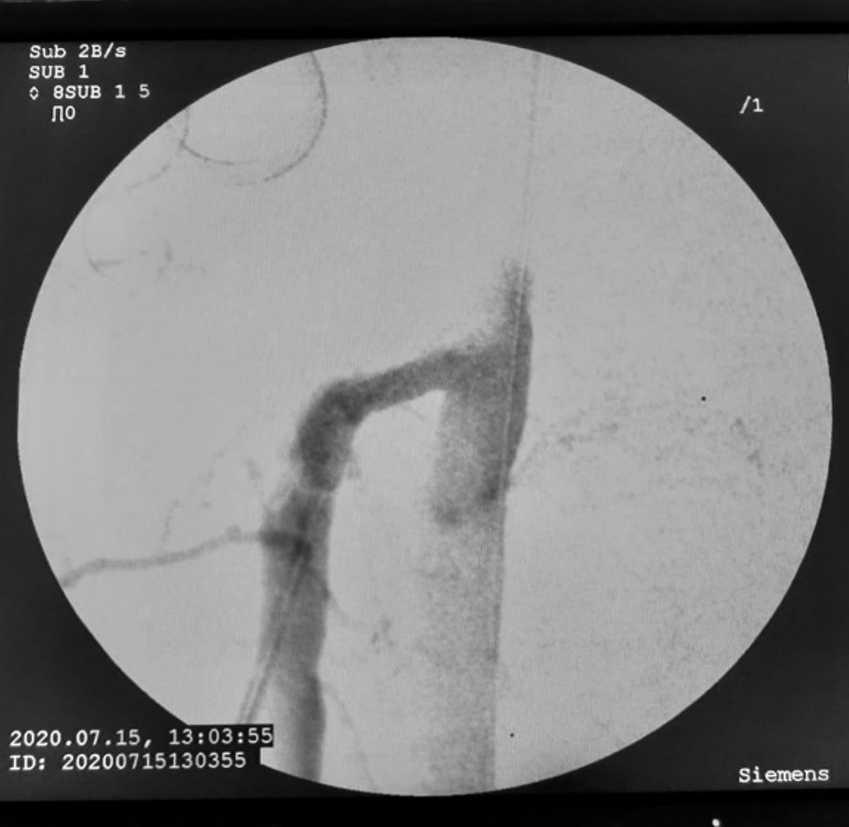
Arteriography after revascularization of the superior mesenteric artery.

**Figure 8:**
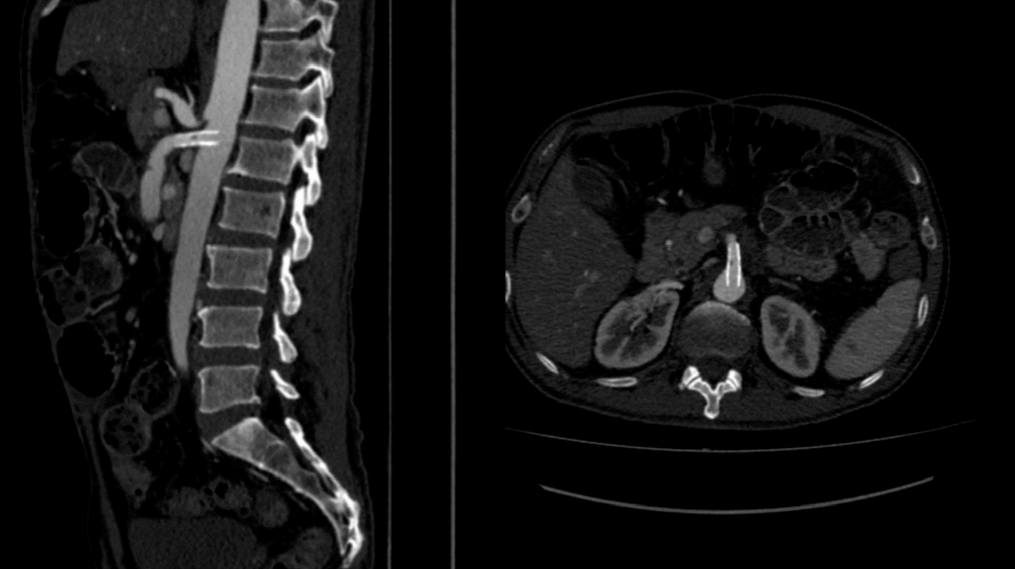
Stent in the proximal portion of the SMA, which is patent.

The patient underwent arteriography, but, because of the complexity of the dissection and the risk of occlusion of branches of the SMA, the decision was taken to conduct open repair. Laparotomy was performed, followed by endarterectomy of the dissected segment of the SMA and arterioplasty with a bovine pericardium patch. On the first postoperative day, the patient complained of a sudden increase in pain and control angiotomography showed occlusion of the distal SMA (Figure 3), suggestive of graft thrombosis, and an intervention was conducted with embolectomy of all of the branches of the SMA and a second arterioplasty with a large pericardium patch (Figure 4). Transoperative Doppler showed 70-80% stenosis at the origin of the SMA*,* which was treated with angioplasty via retrograde puncture of the patch (Figure 5) to stent the SMA at its origin (Figure 6). This was successful and normalized blood flow (Figure 7) and velocity on ultrasound. During the procedure, several areas of ischemia were observed in the intestinal segment, but they were considered viable and a “second look” was scheduled for 24h later to review them. This was conducted as scheduled, revealing an area of necrotic intestine, treated by enterectomy of an approximately 80 cm segment of jejunal loop and enteroanastomosis. After these procedures, the patient progressed well in postoperative recovery, exhibiting improvement of symptoms, and was discharged from hospital with a referral to outpatients follow-up, with monthly consultations and control imaging exams at 3 months (Figure 8).

## DISCUSSION AND CONCLUSIONS

Although rare, and with etiology that is not entirely clear in cases unrelated to trauma or iatrogeny, dissection of the SMA is a serious disease associated with complications such as formation of thrombi, hemorrhages, and mesenteric ischemia.^2^^,^ 8 Hypertension, as observed in the present patient, is not always associated, but can be considered a risk factor, as can smoking. Patients’ clinical presentations can vary and abdominal pains emerge insidiously as a result of intestinal ischemia. In some cases, there is nausea, vomiting, abdominal distension, and even hemodynamic instability.^3^^,^ 4

Angiography is the gold standard on which to base patient treatment decisions, since it enables detailed assessment of the lesion and its severity. There are several types of treatments that can be employed – choices range from conservative treatment^4^ to endovascular surgery or open surgery.^1^^,^ 9 Conservative treatment was initially proposed in the present case and is the most common approach, although it is restricted to patients who are hemodynamically stable and in whom there is no evidence of rupture of the SMA dissection. It is effective in the majority of cases, monitoring patients with imaging exams and a focus on the clinical signs of mesenteric ischemia and vascular supply from the SMA.

Indications for surgery remain controversial and vary depending on clinical presentation, signs of complications, and even recurrence of symptoms. Exploratory laparotomy is indicated if the patient exhibits signs and symptoms of mesenteric ischemia, but different approaches may be complementary, with the option of performing open repair, endovascular repair, or a combination of both.^6^^,^ 10 ^,^ 11 In our case, the attempt at conservative treatment did not resolve the patient’s condition, who needed surgical intervention employing both techniques. The choice between angioplasty followed or not by stenting,^7^^,^ 12 combined with some other method is dependent on the situation, demonstrating that the dynamics of treatment choice are dependent on the presentation of each case and its complexity. In this case, endovascular treatment was used to salvage the failure of the initial open approach, which was identified perioperatively and corrected using a stent. Complications such as intestinal ischemia can continue to progress even after treatment and so it is always necessary to remain alert for signs of this complication or, as was done in this case, schedule a “second look” and perform intestinal resection if needed.

Since SMA dissection is very often difficult to diagnose^13^ and there is still no consensus on the best treatment in each case,^8^^,^ 14 clinical signs and patient follow-up are essential for a favorable outcome. When necessary, the surgical approach most appropriate for each case should be chosen, whether open repair, endovascular repair, or a combination of both.
