# Supplementary material for: Combination treatment for superior mesenteric artery dissection: therapeutic challenge
Source: J Vasc Bras. 2022 Mar 7;21:e20210157. doi: 10.1590/1677-5449.210157 (PMC8958437; doi:10.1590/1677-5449.210157)
Supplement: Translation

# Translation: Tratamento combinado na dissecção de artéria mesentérica superior: desafio terapêutico

**Authors:** Guilherme Borgo Ficagna, Cristiano do Carmo Galindo, Jean Paulo Niero Mazon, Gustavo Galvan Debiasi, Amanda Bogo Vargas, Laura Sahd Bernz

**Affiliations:** 1 Hospital Maternidade Marieta Konder Bornhausen – HMKB, Itajaí, SC, Brasil.; 2 Universidade do Vale do Itajaí – UNIVALI, Itajaí, SC, Brasil.; 3 Hospital de Clínicas de Porto Alegre – HCPA, Porto Alegre, RS, Brasil.

**Keywords:** artéria mesentérica superior, dissecção, endarterectomia, angioplastia

## Abstract

A dissecção da artéria mesentérica superior é uma causa rara de dor abdominal, com quadro clínico variável. Seu diagnóstico é difícil, e não existe consenso sobre suas opções terapêuticas; elas variam em torno de tratamento conservador, correção aberta, endovascular ou combinada. Descrevemos o caso de um homem de 45 anos com dissecção isolada da artéria mesentérica superior, com quadro de dor abdominal persistente após tentativa de tratamento conservador. Ele foi submetido à revascularização cirúrgica aberta devido à localização e complexidade da dissecção. O tratamento com endarterectomia, arterioplastia com remendo de pericárdio bovino e acesso retrógrado para abertura da mesentérica com stent foi realizado com sucesso. A angina abdominal foi totalmente resolvida após estabilização do quadro. A combinação de abordagem aberta e endovascular deve ser considerada como terapia para casos de dissecção complexa isolada da artéria mesentérica superior.

## INTRODUÇÃO

A dissecção da artéria mesentérica superior (AMS) é uma causa rara de dor abdominal e de difícil diagnóstico^1^^,^ 2. A tomografia computadorizada protagoniza o método mais reconhecido para a detecção da doença, uma vez que evidencia a lesão na maioria das vezes e auxilia no planejamento da abordagem^3^^,^ 4. As opções terapêuticas para a dissecção de AMS são variáveis e vão do tratamento conservador ao reparo cirúrgico aberto^5^, endovascular ou combinado^6^^,^ 7, dependendo da gravidade do caso. O projeto foi aprovado pelo Comitê de Ética e Pesquisa (CAAE 47854621.0.0000.0120, parecer consubstanciado 4.875.653).

## PARTE I: CASO CLÍNICO

Paciente masculino de 45 anos, hipertenso e ex-tabagista, com quadro de dor abdominal difusa e recorrente com 30 dias de evolução, com piora no último dia, sem demais queixas associadas. Ele mantinha sinais vitais estáveis e, ao exame físico abdominal, apresentava dor leve à palpação, sem sinais de irritação peritoneal. Foi submetido a tomografia com contraste, que evidenciou dissecção isolada da AMS (Figura 1), mantendo perfusão de todos os seus ramos. Nesse primeiro momento, optou-se por internação hospitalar e controle da dor com sintomáticos e antiagregação. Como houve melhora dos sintomas com analgesia e os exames laboratoriais se mantiveram sem alterações durante toda internação, o paciente recebeu alta após 5 dias para manter tratamento e seguimento ambulatorial. Retornou ao hospital no dia seguinte da alta hospitalar referindo piora da dor, sendo novamente submetido a tomografia com contraste, que não evidenciou alteração em relação ao exame anterior. O paciente reinternou pelo quadro álgico e recebeu alta após 7 dias de internação, estando clinicamente assintomático, ainda em uso de antiagregantes. No dia seguinte, retornou novamente ao hospital com quadro de piora da dor, e a imagem de controle evidenciou ausência de fluxo vascular nos ramos distais da AMS (Figura 2), sugerindo tromboembolismo. Nesse momento, o próximo passo seria definir a conduta terapêutica entre as possibilidades de tratamento, sendo elas reparo aberto, endovascular ou combinado.

**Figura 1:** Dissecção isolada de artéria mesentérica superior mantendo perfusão de todos os seus ramos.

**Figura 2:** Presença de *flap* intimal na artéria mesentérica superior sugestivo de dissecção e ausência de fluxo vascular nos ramos distais, sugerindo tromboembolismo.

## PARTE II: TERAPÊUTICA

O paciente foi submetido a arteriografia, porém, devido à complexidade da dissecção e ao risco de oclusão de ramos da AMS, optou-se pela correção cirúrgica aberta. Foi realizada a laparotomia, seguida de endarterectomia do segmento de dissecção da AMS e de arterioplastia com remendo de pericárdio bovino. No primeiro dia de pós-operatório, o paciente evoluiu com piora súbita da dor, e a angiotomografia de controle evidenciou oclusão da AMS na porção distal (Figura 3), sugerindo trombose do enxerto, sendo submetido a nova intervenção com embolectomia de todos os ramos da AMS, além de nova arterioplastia com extenso remendo de pericárdio (Figura 4). Foi realizado Doppler transoperatório, que evidenciou estenose de 70-80% na origem da AMS*,* sendo, então, realizada angioplastia através de punção retrógada do remendo (Figura 5) com implante de stent na origem da AMS (Figura 6) com sucesso e normalização do fluxo (Figura 7) e velocidade ao ultrassom. Observou-se, no transoperatório, várias áreas de isquemia do segmento intestinal, porém viáveis, sendo programado *second look* após 24h para revisão. Conforme programado, realizou-se, então, nova abordagem, que evidenciou área de sofrimento de alça, seguida de enterectomia segmentar de aproximadamente 80 cm de alça de jejuno e enteroanastomose. Após as abordagens, o paciente teve boa evolução na recuperação pós-operatória, apresentando melhora dos sintomas e recebendo alta hospitalar com encaminhamento para seguimento ambulatorial, sendo realizadas consultas mensais e imagens de controle após 3 meses (Figura 8).

**Figura 3:** Artéria mesentérica superior pérvia na porção proximal com falha de enchimento abrupta na porção distal, sugerindo trombose do enxerto.

**Figura 4:** Remendo da artéria mesentérica superior.

**Figura 5:** Punção retrógrada do remendo da artéria mesentérica superior.

**Figura 6:** Arteriografia da artéria mesentérica superior.

**Figura 7:** Arteriografia após revascularização da artéria mesentérica superior.

**Figura 8:** Stent na porção proximal da AMS e sua perviedade.

## DISCUSSÃO E CONCLUSÃO

Apesar da raridade e da etiologia não totalmente esclarecida nos casos não associados a trauma ou iatrogênica, a dissecção da AMS é uma doença grave associada a complicações como formação de trombos, hemorragias e isquemia mesentérica^2^^,^ 8. A hipertensão, como no paciente relatado, nem sempre está associada, mas pode ser considerada fator de risco, bem como o tabagismo. A clínica do paciente é variável, podendo a dor abdominal estar presente de forma insidiosa como resultado de isquemia intestinal. Em alguns casos, há presença de náuseas, vômitos, distensão abdominal e até mesmo instabilidade hemodinâmica^3^^,^ 4.

A angiografia é o padrão ouro para a decisão do tratamento do paciente, uma vez que ela permite a detalhada avaliação da lesão e de sua gravidade. Há uma diversidade de tratamentos a se seguir – a escolha diverge entre tratamento conservador^4^, endovascular ou cirurgia aberta^1^^,^ 9. O tratamento conservador, como foi proposto inicialmente para o caso, é o mais comum, sendo reservado para os casos em que os pacientes se apresentam com estabilidade hemodinâmica e em que não há evidência de ruptura da dissecção da AMS. Esse tratamento é efetivo na maioria dos casos, mantendo-se acompanhamento com exames de imagem e foco nos sinais clínicos de isquemia mesentérica e no suprimento vascular da AMS.

As indicações para a cirurgia ainda são controversas e variam de acordo com a apresentação clínica, sinais de complicação e até mesmo recorrência dos sintomas. A laparotomia exploratória está indicada caso o paciente apresente sinais e sintomas de isquemia mesentérica, entretanto as abordagens podem ser complementares, sendo o reparo aberto, endovascular ou combinado^6^^,^ 10 ^,^ 11. No nosso caso, a tentativa de tratamento conservador não obteve resolução do quadro, sendo necessária intervenção cirúrgica em que utilizamos ambas as técnicas. A opção entre angioplastia seguida ou não da colocação de stent^7^^,^ 12 associada a algum outro método depende de cada situação, demonstrando que a dinâmica para escolha de tratamento depende da apresentação de cada caso e de sua complexidade. Neste caso, o tratamento endovascular foi usado como resgate de uma falha no tratamento aberto inicial, identificada no transoperatório e corrigida com uso de stent. Complicações como isquemia intestinal podem cursar com evolução mesmo após a terapêutica; por isso, sempre é necessária a atenção aos sinais dessa complicação ou, bem como foi optado neste caso, um *second look* e ressecção intestinal quando houver necessidade.

Como a dissecção de AMS é muitas vezes de difícil diagnóstico^13^ e ainda não existe consenso sobre o melhor tratamento para cada caso^8^^,^ 14, os sinais clínicos e o acompanhamento do paciente são essenciais para um desfecho favorável. Quando necessário, deve ser indicada a abordagem cirúrgica mais adequada para cada caso, seja por reparo aberto, endovascular ou combinado.
